# The altered hypothalamic network functional connectivity in diminished ovarian reserve and regulation effect of acupuncture: a randomized controlled neuroimaging study protocol

**DOI:** 10.3389/fendo.2025.1598943

**Published:** 2025-08-08

**Authors:** Feng Gao, Yang Yu, Fuwei Wang, Yigong Fang

**Affiliations:** 1 Institute of Acupuncture and Moxibustion, China Academy of Chinese Medical Sciences (CACMS), Beijing, China; 2 Department of Acupuncture and Tuina, School of Medicine of the Affiliated Sir Run Run Shaw Hospital, Zhejiang University, Hangzhou, China

**Keywords:** diminished ovarian reserve, rs- fMRI, hypothalamic-pituitary-ovarian axis, functional connectivity, acupuncture

## Abstract

Diminished ovarian reserve (DOR) is characterized by a decrease in the quantity and quality of oocytes, leading to reduced chances of natural conception and a poorer response to fertility treatments. Along with these reproductive challenges, DOR often causes psychological symptoms such as depression, anxiety, and sleep disturbances, which negatively affect overall well-being and quality of life. Acupuncture has been proposed as a promising complementary therapy for DOR, but the mechanisms through which it exerts its effects are not yet fully understood. This study aims to investigate the effects of acupuncture on ovarian function, psychological well-being, and the central nervous system in women with DOR. We will recruit 42 women with DOR and 21 healthy controls (HCs), randomly assigning DOR patients to receive either verum acupuncture (VA) or sham acupuncture (SA) for 12 weeks. Ovarian function will be assessed using Anti-Müllerian hormone (AMH), antral follicle count (AFC), and follicle-stimulating hormone (FSH). Psychological well-being will be evaluated using the Self-Rating Anxiety Scale (SAS), Self-Rating Depression Scale (SDS), and Self-Rating Scale of Sleep (SRSS). To explore the neurological effects, resting-state functional connectivity (rsFC) of the hypothalamus will be assessed using functional magnetic resonance imaging (fMRI). This research aims to clarify how acupuncture affects the central nervous system, hormonal regulation, and ovarian function in women with DOR. The findings may provide valuable insights for developing evidence-based acupuncture protocols that can improve both reproductive outcomes and quality of life for women with DOR.

## Introduction

Ovarian function is crucial for female fertility, as it produces oocytes and secretes hormones such as estrogen and progesterone (1). Diminished ovarian reserve (DOR), characterized by reduced oocyte quantity and/or quality, presents a significant challenge in reproductive medicine, impacting fertility outcomes. While the concept is widely recognized, its precise definition remains debated (2). Clinically, DOR is often diagnosed by markers such as antral follicle count (AFC) < 5, follicle-stimulating hormone (FSH) > 10 IU/L, or AMH< 0.5–1 ng/mL (3). These physiological changes can manifest as menstrual irregularities (e.g., shortened periods, hypomenorrhea, amenorrhea), infertility, and even perimenopausal symptoms like hot flashes, insomnia, decreased libido, and potentially progress to premature ovarian insufficiency or failure (POI/POF) significantly affecting a woman’s reproductive health and overall well-being (4, 5). DOR also increases the risk of cardiovascular disease, osteoporosis, and mental health issues like insomnia, anxiety and depression compared to women experiencing normal menopause (6). Studies show significantly higher rates of depression and perceived stress in women with the loss of ovarian function as well, impacting their quality of life and productivity, and highlighting the substantial psychological burden associated with DOR (7). The underlying causes of DOR are complex and multifactorial, potentially involving genetic predisposition, oxidative stress, mitochondrial dysfunction, inflammation, and fibrosis, all of which impact the ovarian microenvironment (7, 8). This complexity, coupled with the limitations of current diagnostic markers for early detection, makes effective prevention and treatment of DOR challenging.

DOR is a heterogeneous disorder with diverse clinical manifestations that not only impair fertility but also cause menstrual irregularities and perimenopausal symptoms (e.g., insomnia, anxiety, depression, and cognitive dysfunction) (9). Compelling evidence reveals a bidirectional relationship between DOR and psychological distress - approximately 50% of DOR patients exhibit significant depressive symptoms, anxiety, and emotional dysregulation, which may exacerbate DOR progression through hypothalamic-pituitary-gonadal axis dysfunction (10). This neuroendocrine-psychological interplay underscores the mutual reinforcement between DOR and mood disorders, with potential central nervous system consequences. Previous studies have suggested that abnormalities in the hypothalamic-pituitary-ovarian (HPO) axis may be a potential underlying cause of DOR (11). Dysregulation at any point of this axis, whether at the hypothalamic, pituitary, or ovarian level, can disrupt the normal hormonal signaling required for optimal ovarian function, leading to a reduction in the quantity and quality of oocytes (12). Such dysfunction may result from various factors, including endocrine disorders, genetic predispositions, or environmental influences, which can ultimately contribute to the pathogenesis of DOR (13). HPO axis is critical for the maintenance of mental and physical health (14). Understanding these disruptions in the HPO axis may offer new insights into the mechanisms driving DOR and pave the way for more effective diagnostic and therapeutic approaches. In recent decades, the advent of noninvasive functional magnetic resonance imaging (fMRI) has provided unprecedented insights into brain activity, enabling the exploration of central physiological processes with remarkable precision. This advanced imaging technique has revolutionized our ability to investigate the dynamic neural mechanisms underlying a wide range of mental, physical, and emotional functions, thus significantly enhancing our understanding of brain function in both health and disease (15–17).

DOR patients might suffer from chronic estrogen deficiency, hormone replacement therapy (HRT) is one of the most widely used treatment (18). This treatment approach seeks to relieve symptoms and restore hormone levels to a physiologically normal range, but it also comes with potential drawbacks, such as increase the risk of ovarian cancer (19). Platelet-rich plasma (PRP) is another promising treatment that has shown the potential to promote endometrial and follicle growth for restoring female reproductive and endocrine function, while the results shows that it do not improve IVF outcomes in women under 38 and lack of standardization (20, 21). Given this context, there is an urgent need for an effective treatment for DOR. Acupuncture is a traditional Chinese medical (TCM) approach involves treating patients by stimulating specific acupoints on the body to regulate the flow of Qi and blood within the meridian system. For decades, acupuncture has found extensive application in the treatment of a diverse array of physical or mental diseases, encompassing conditions like infertility, ovarian insufficiency, menstrual cycle irregularity, insomnia, and depression (6, 22–24). Acupuncture has been widely used to treat DOR, and has demonstrated curative effects (25, 26). Also, acupuncture could modulate homeostasis of HPO axis (27). However, due to a lack of high-quality evidence supporting its use and insufficient elucidation on the mechanisms underlying acupuncture’s effects.

In another study protocol designed to evaluate the effects of acupuncture on menstrual regulation and pregnancy enhancement in patients with DOR, enrolled only DOR patients with verum acupuncture (VA) treatment and compared the related changes in brain functional network and other indicators of ovarian function (28), which didn’t consider the placebo effect, and its potential clinical relevance. Sham acupuncture (SA) serves as a placebo control in acupuncture clinical trials, designed to elucidate both specific and nonspecific components pertinent to acupuncture therapy (29, 30). There is still controversy regarding site-specificity in acupuncture, some authors have suggested that SA interventions were not totally inert, often being associated with moderately large non-specific effects (31). When VA is compared with SA, different cerebral responses are observed, which may explain the favorable effects of VA in clinical studies on pain treatment (32). Various approaches have been employed to mimic SA, some studies have adopted simulated needling procedures, which potentially exert effects akin to acupressure, while others have utilized superficial needling at non-acupoints or incorrect acupoints based on traditional theories (29). Penetrating SA may induce additional clinical treatment effects and influence brain function, potentially confounding the results. Therefore, in this study, we will use non-penetrating sham devices to minimize nonspecific effects and more accurately simulate the specific clinical efficacy of VA.

In this study, we aim to assess the impact of acupuncture on HPO axis-related brain function and the relationship between homeostasis of HPO axis and sleep quality, depression, and anxiety in patients with DOR.

## Materials and methods

### Study design

**Figure 1:**
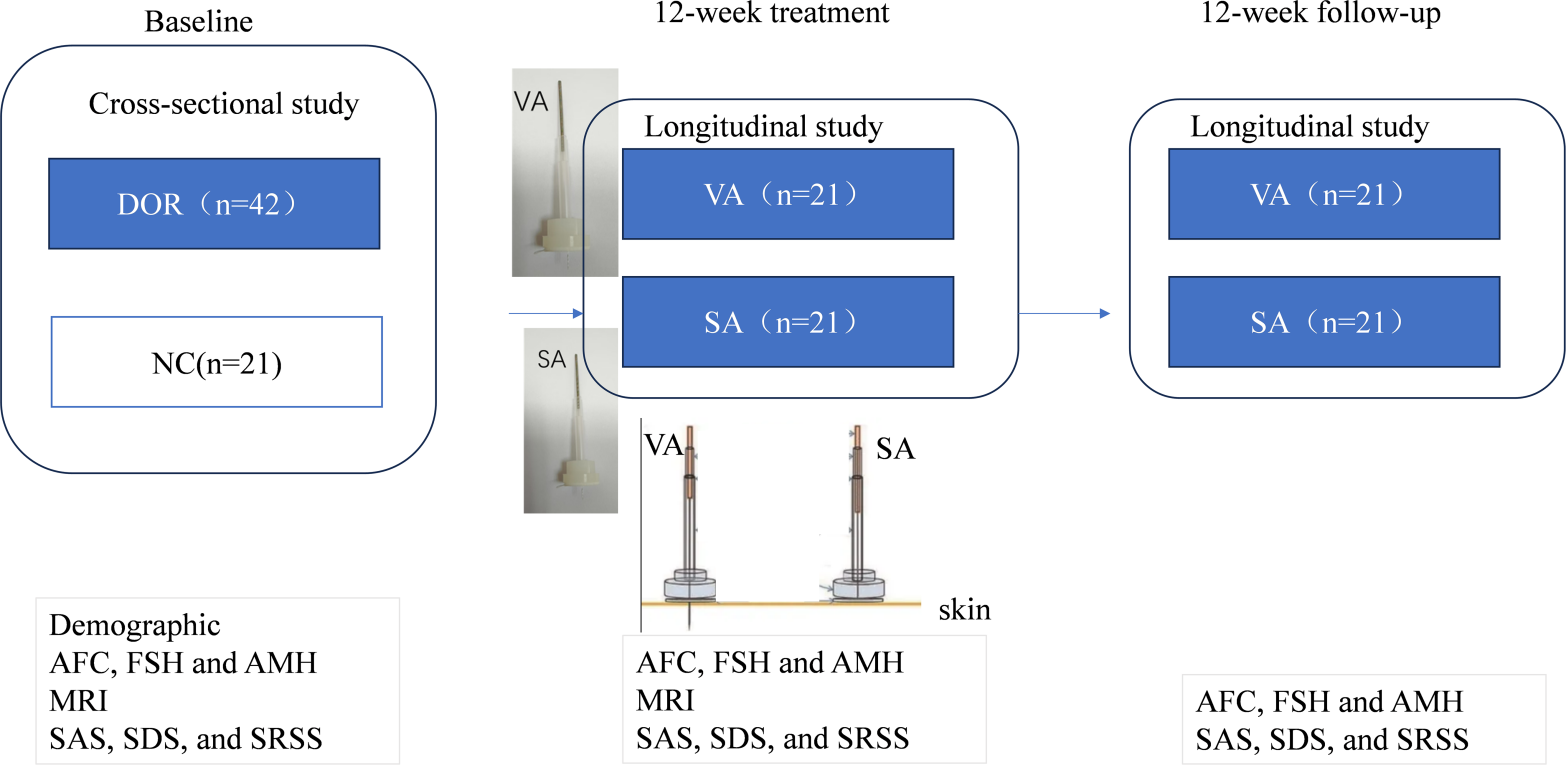
1 Trial flow chart.

This study will consist of two experiments: a cross-sectional experiment and a longitudinal experiment (**Figure 1**). The cross-sectional experiment will examine brain functional connectivity (FC) in patients with DOR by comparing seed-based FC between DOR patients and healthy controls (HCs). The longitudinal experiment will investigate the clinical efficacy of acupuncture and its underlying neurological mechanisms by comparing FC before and after treatment. Additionally, we will compare the differences in FC and clinical symptoms, such as ovarian function, anxiety, depression, and sleep disturbances, between the VA and SA groups following treatment. Finally, we plan to analyze the relationship between changes in FC and clinical symptoms. This will allow us to investigate whether acupuncture can modulate hypothalamic FC and subsequently improve clinical symptoms in DOR patients. The longitudinal acupuncture experiment was approved by the Ethics Committee of Sir Run Shaw Hospital Zhejiang University School of Medicine (2025YL0012) and submitted to the International Traditional Medicine Clinical Trial Registry (ITMCTR2025000576). All participants will be provided written informed consent before the initiation of any study procedures. The study adhered to the CONSORT guidelines for reporting

### Participants

We plan to recruit 42 patients with DOR and 21 healthy female volunteers of childbearing age through WeChat, poster postings, and other channels. A dedicated staff member will be responsible for patient recruitment and will meticulously record each participant’s information, which will be documented in the Case Report Form (CRF). The recruitment materials will include detailed contact information. This study adheres to the principles outlined in the Declaration of Helsinki.

Inclusion criteria for healthy controls:

- Age between 20 and 40 years (inclusive).
- Normal gynecological ultrasound, normal hormone levels, and regular menstrual flow with normal color and quantity.
- No severe heart, liver, kidney, or hematologic system disorders.
- Right-handed.
- Voluntary participation in the trial and signed informed consent.

The inclusion criteria for DOR are similar to our previous design (23):

- Age between 20 and 40 years (inclusive).
- Diagnosis of DOR based on the 2016 POSEIDON criteria and the 2020 expert consensus on the treatment of low-prognosis populations in assisted reproductive technology using the Delphi method in China.
- AMH level < 1.2 ng/ml.
- AFC < 5 (Note: AFC should be measured on days 2–3 of the menstrual cycle, with at least a 4-week interval between two measurements).
- Fertility desire, with plans for natural conception or, as assessed by a reproductive specialist, plans to undergo controlled ovarian stimulation with an antagonist protocol in the next *in vitro* fertilization (IVF) and embryo transfer cycle.
- Right-handed.
- Voluntary participation in the trial and signed informed consent.

Exclusion criteria:

- Coexisting conditions that affect fertility, including congenital reproductive organ abnormalities, untreated endometrial polyps, untreated uterine infections, untreated tubal hydrosalpinx, thin endometrium, uterine fibroids, endometriosis, or other organic diseases.
- Coexisting polycystic ovary syndrome (PCOS).
- Coexisting untreated hyperprolactinemia, hyperandrogenism, thyroid dysfunction, chronic adrenal insufficiency, or other metabolic and endocrine disorders.
- Acupuncture treatment for fertility enhancement received within the last three months.
- Plans for *in vitro* fertilization (IVF-ET) within 2 months after enrollment.
- Vulnerable populations other than illiterate individuals, including those with mental illness, cognitive impairments, critically ill patients, pregnant women, etc.

Regular reminders via WeChat or telephone will be used to improve participant adherence. If any of the following events occur in the enrolled patients, their intervention will be terminated forcibly:

- Serious complications or other significant illnesses that require emergency measures.
- Pregnancy;
- Withdrawal of informed consent for any reason. There is no post- trial intervention or care for patients in this trial.

No treatment will be given to healthy group, only one fMRI, laboratory test and neuro-psychological test will be performed at baseline.

### Acupuncture interventions

Eligible participants will be randomly allocated into the VA or SA group at a 1:1 ratio via the web-based response system of the Central Randomisation System for Clinical Research. Stratified randomization will be applied, with age (35 years) as the stratification factor. Unblinding will not be required during the study as it will not have clinical relevance to treatment decisions. The randomization plan will be generated using the “Proc plan” procedure in SAS 9.3 statistical software (SAS Institute Inc., Cary, NC, USA), and all related parameters set during the randomization process will be stored in a blinded database. When eligible participants are enrolled, the randomization number will be assigned by the responsible personnel or researcher through phone or online access to the central randomization system. The random distribution cards will contain random numbers, serial numbers, and groups, packed in opaque numbered envelopes. The envelope will be unsealed following study enrolment. Owing to the particularity of acupuncture research, it is difficult to blind the acupuncture operators. As such, fake acupuncture devices and separate treatment rooms will be used for each patient to blind them as much as possible. Moreover, the “three separations strategy” will be strictly adhered to by researchers, operators, and statisticians throughout the research process. Efficacy evaluation, data analysis, and statistics will be completed by third parties blinded to the groupings.

Acupuncture or sham acupuncture will be administered by certified acupuncturists and have received prior standardized training in operation. Both groups chose the same acupoints, which is same to our previous protocol (26), including two sets of acupoints (group A and group B) comprising 14 selected points will be manually applied to patients, alternately, starting with group A (number of needle insertions per patients per session: 6 or 20). Based on the prior studies, the “Fertility-Menstruation Regulating Thirteen Acupuncture Points” was chosen for this trial. Acupoints of group A include Baihui (GV20), Shenting (GV24), bilateral Benshen (GB13), Zhongwan (CV12), bilateral Tianshu (ST25), Guanyuan (CV4), bilateral Zigong (EX- CA1), bilateral Dahe (KI12), bilateral Zusanli (ST36), bilateral Sanyinjiao (SP6) and bilateral Taichong (LR3). Group B consists of three acupoints: bilateral Shenshu (BL23), bilateral Zhongliao (BL33) and bilateral Taixi (KI3). The locations of each of the acupoints according to the WHO Standard Acupuncture Point Locations in the Western Pacific Region are listed in **Table 1**.

**Table 1:** Locations of acupoints.

Acupoint	Location	Insert angle	Insert depth
GV20	On the head, 5 proportional bone (skeletal) cun (B-cun) superior to the anterior harline, on the anterior median line	0°	10–20 mm
GV24	On the head, 0.5 B-cun superior to the anterior hairline, on the anterior median line	15°	10–20 mm
GB13	On the head, 0.5 B-cun superior to the anterior hairline, 3 B-cun lateral to the anterior median line	15°	10–20 mm
CV12	On the upper abdomen, 4 B-cun superior to the centre of the umbilicus, on the anterior median line	90°	30–40 mm
ST25	On the upper abdomen, 2 B-cun lateral to the centre of the umbilicus	90°	30–40 mm
CV4	On the lower abdomen, 3 B-cun inferior to the centre of the umbilicus, on the anterior median line	90°	30–40 mm
EX-CA1	On the lower abdomen, at the same level as Zhongji (CV3), 3 B-cun lateral to the anterior median line	90°	30–40 mm
KI12	On the lower abdomen, 4 B-cun inferior to the centre of the umbilicus, 0.5 B-cun lateral to the anterior median line	90°	30–40 mm
ST36	On the anterior aspect of the leg, on the line connecting Dubi (ST35) with jiexi (ST41), 6 B-cun inferior to ST35	90°	30–40 mm
SP6	On the tibial aspect of the leg, posterior to the medial border of the tibia, 3 B-cun superior to the prominence of the medial malleolus	90°	30–40 mm
LR3	On the dorsum of the foot, between the first and second metatarsal bones, in the depression distal to the junction of the bases of the two bones, over the dorsalis pedis artery	90° [obliquely towards Yongquan (KI1)]	10–20 mm
BL23	In the lumbar region, at the same level as the inferior border of the spinous process of the second lumbar vertebra(L2),1.5 B-cun lateral to the posterior median line	90°	30–40 mm
BL33	In the sacral region, in the third posterior sacral foramen	90°	60–70 mm
KI3	On the posteromedial aspect of the ankle, in the depression between the prominence of the medial malleolus and the calcaneal tendon	90°	30–40 mm

The patients were asked to lie on their backs or stomach (depends on group A or B) in a quiet position, exposed to acupuncture. For VA group, after 75% alcohol disinfection, the Hwato^®^ (Huatuo brand made in Suzhou Medical Appliance Factory, Jiangsu, China) disposable needle (Φ=0.25*25 mm, Φ=0.25*40 mm, Φ=0.30*75 mm) is used in conjunction with the Park Sham Acupuncture Device (PSD, DongBang acupuncture company) (30). Expose the tip of the needle, apply a suitable needle insertion method, and attach the park device to the skin. Depending on the muscular layer depth of the acupuncture points, we adjusted the needle insertion depth until the patient experienced the “deqi” sensation. Needle manipulation techniques such as lifting, thrusting or twirling will be applied to promote ‘deqi’ (a sensation experienced by patients at the acupoint location, characterized by soreness, numbness, heaviness, and distention, etc). Acupuncture needles for VA will be inserted perpendicularly into a depth of 10–20 mm for GV20, GV24 and GB13, obliquely towards Yongquan (KI1) into a depth of 10–20 mm for LR3, deeply into a depth of 60–70 mm into the third posterior sacral foramina for BL33, and perpendicularly into a depth of 30–40 mm for the remaining acupoints. Needle retention time will last for 20 min per session, once every other day, 3 sessions a week for 12 weeks, totaling 36 treatment sessions.

The SA group will receive a non-inserted acupuncture using the sham needle supported by the PSD. This needle has a retractable needle shaft and a blunt tip, they could not penetrate the skin (33). We gently placed the sham needle and PSD on the skin. The sham needle is then left no more manipulated to avoid any further physiological effects, thereby minimizing the potential for additional effects (34). The acupoints is same to the VA group. At the end of the treatment, the acupuncturist also used a dry cotton ball to press the acupoints so that patients could feel the withdrawal of ‘real’ needles (35).

At the conclusion of the trial, all participants were asked to complete a blinding assessment questionnaire containing the following question: “Which group do you believe you were assigned to during the study?” The response options were: A) VA (treatment group), B) SA (control group), or C) Uncertain. This assessment served three primary purposes: First, to quantify blinding efficacy by calculating the percentage of correct versus incorrect guesses. Second, to validate whether the sham acupuncture procedures had successfully masked group allocation. Finally, to assess any potential bias in self-reported outcomes, particularly for psychological scales.

During the trial period, all participants were explicitly instructed to refrain from any additional interventions, including moxibustion, cupping, and herbal remedies, that could potentially influence the study outcomes.

### Clinical outcome measurement

The AFC will be measured via transvaginal ultrasonography on days 2–5 of the menstrual cycle. AFC, along with serum levels of basal FSH and AMH, will be assessed at three time points: baseline (week 0), post-treatment (week 12), and 12-week follow-up (week 24).

The neuro-psychological test such as SAS, SDS, and SRSS will be used for clinical assessments at three time points: baseline (week 0), post-treatment (week 12), and 12-week follow-up (week 24).

The time points for each test in two group is listed in **Table 2**.

**Table 2:** Time points for each test in two groups .

Measurements	Baseline		12 weeks		24 weeks	
	DOR	HC	DOR	HC	DOR	HC
AFC, FSH, AMH	✓	✓	✓		✓	
AFC	✓		✓			
SAS, SDS, and SRSS	✓	✓	✓		✓	
MRI	✓	✓	✓			

Diminished ovarian reserve, DOR; healthy control, HC; anti-Müllerian hormone, AMH; antral follicle count, AFC; follicle-stimulating hormone, FSH; self-rating anxiety scale, SAS; self-rating depression scale, SDS; self-rating scale of sleep, SRSS; magnetic resonance imaging, fMRI.

### fMRI data acquisition

All DOR patients received two separate resting-state fMRI scanning, before and after the 12 weeks acupuncture treatment course, respectively. The healthy controls participated only in one resting-state scanning as baseline control. For all participants, fMRI scanning should be done at least 3 day away from their menstrual period.

In this study, 3.0 T MRI scanner (Siemens, Sonata, Germany) at the Sir Run Shaw Hospital, was used to perform cranial scans on all subjects. (1) T1-weighted imaging (T1WI): horizontal axis position, TR = 600 ms, TE = 10 ms, slice thickness = 4 mm, slice gap = 1.5 mm, field-of-view (FOV) = 240 mm × 240 mm, matrix = 256×256. (2) Resting-state fMRI sequence and parameters: acquired with gradient echo-echo planar imaging (GRE-EPI) sequence, TR = 2000 ms, TE = 30 ms, slice thickness = 4 mm, slice gap = 1.2 mm, FOV = 220 mm × 220 mm, matrix = 64 × 64, flip angle = 90°, 200 time points in total, 35 slices scanned continuously. The entire brain will be scanned, and during the procedure, participants will be instructed to lie supine with their eyes open, while being asked to refrain from focusing on any specific thoughts.

### Incidence of adverse events

All adverse events (AEs) will be recorded and assessed. The principal investigator and ethics committee will determine whether the participants should withdraw from the trial. AEs contain any unfavorable but mild signs, symptoms, or feelings. AEs will be recorded in detail in the case report forms (CRFs). Acupuncture is a minimally invasive treatment, and its adverse events cannot be completely avoided.

### Sample size calculation

This is an exploratory study, and according to the highly cited experimental fMRI studies had a single group of participants and these studies had median sample size of 12, the sample size of 12 cases in each group and 20 to 30 cases has become the “minimum requirement” and most choices for acupuncture imaging confirmatory studies. We have expanded the sample size to 21 cases per group (36).

### Demographic and clinical data analyses

Demographic and clinical outcome analyses will be conducted using SPSS 22.0 software. In the cross-sectional study, two-sample t-tests and Chi-square tests will be employed to compare baseline characteristics between the DOR patient group and HCs. In the longitudinal study, paired t-tests will be used to compare changes in clinical symptoms (SAS, SDS, and SRSS scores) and laboratory test results (AFC, FSH, and AMH) within each group before and after treatment. Subsequently, a two-way analysis of variance (ANOVA), with time as the within-subject factor, will be applied to examine differences in clinical symptoms between the VA and SA groups before and after treatment. A *p*-value of < 0.05 will be considered statistically significant.

### MRI data preprocessing

Preprocessing of MRI images will be conducted using the DPARSF (http://rfmri.org) and SPM 12 (www.fil.ion.ucl.ac.uk/spm) toolkits within MATLAB 2014b (MathWorks, Natick, MA). The preprocessing pipeline will include the following steps: (1) removal of the first 10 volumes; (2) slice timing correction; (3) realignment of images to correct for head motion; (4) co-registration of T1-weighted images with functional images; (5) normalization of T1 images to Montreal Neurological Institute (MNI) space, followed by segmentation into gray matter, white matter, and cerebrospinal fluid; (6) spatial smoothing of the images using an isotropic Gaussian kernel with a full width at half maximum (FWHM) of 6 mm; (7) temporal bandpass filtering (0.01-0.08 Hz); and (8) regression of nuisance signals, including global mean, white matter, cerebrospinal fluid signals, and six motion parameters. Considering the aim is to estimate the relationship between ovarian and brain and the hypothalamic-pituitary-ovarian (HPO) axis, the region of interest (ROI) will be selected as the hypothalamus. For static functional connectivity (FC) analysis, Pearson’s correlation coefficients between the reference time series of each seed and the time series of all brain voxels will be computed. These coefficients will then be transformed into z-scores using Fisher’s z-transformation to enhance normality (24). Two-sample t-tests will be conducted to identify significant differences in FC between the DOR and healthy control (HC) groups, with age and mean framewise displacement (FD) as covariates. Moreover, Kisspeptin is a key regulator of gonadotropin secretion and is primarily located in the rostral preoptic area and the infundibular nucleus of the human hypothalamus (37). In this study, we will refer to the segmentation method described in Ogawa (38) to perform subregion segmentation of the hypothalamus in our subjects, with a particular focus on the rostral preoptic area and the infundibular nucleus, as well as the whole-brain FC. For the longitudinal analysis, paired t-tests will be used to compare within-group statistical maps, while a two-way analysis of variance (ANOVA) will be applied to compare between-group statistical maps. Multiple comparisons will be corrected for using a threshold of *p* < 0.05 at the voxel level and *p* < 0.05 at the cluster level, with false discovery rate (FDR) correction.

A conjunction analysis will be performed to investigate whether the altered hypothalamic functional connectivity (FC) in DOR patients is influenced by acupuncture treatment. This analysis will compare the FC change maps in DOR patients with the treatment effects observed in the VA and SA groups, respectively. Additionally, to examine the relationship between clinical outcomes and FC changes, correlation analyses will be conducted between the significantly altered hypothalamus-related FCs and the corresponding clinical improvements in the VA group.

## Discussion

This study aims to explore the efficacy of acupuncture treatment for DOR and its underlying brain mechanisms. The objective is to gain a deeper understanding of the central nervous system mechanisms involved in DOR and to evaluate the effectiveness of acupuncture in treating DOR, along with its potential mechanisms of action. By examining the relationships between brain function, ovarian reserve-related indicators, and neuropsychological scales in DOR patients and HCs at baseline, as well as the changes in brain function before and after treatment in DOR patients, this study will investigate the impact of both VA and SA on brain function. Ultimately, the research seeks to elucidate the potential mechanisms through which acupuncture may influence brain function and ovarian reserve in DOR patients.

According to TCM principles, different researchers may have varying views on the underlying mechanisms. ZHAN’s team identified kidney deficiency, liver-Qi stagnation, and dysfunction of the Thoroughfare and Conception vessels as the most relevant factors (39). Based on our clinical experience, our team primarily attributes the condition to kidney deficiency, dysfunction of the Thoroughfare and Conception vessels, and the reciprocal relationship between emotional imbalance and the disease. We selected 14 specific acupuncture points based on these TCM principles and our previous study; to target the dysfunctions of Kidney, Thoroughfare Vessel and Conception Vessel associated with DOR. Based on the pathways of the meridians and the close connection between the primary disease and its manifestations, particular emphasis is placed on the importance of the Governor, Conception, and Thoroughfare vessels in gynecological conditions. Fourteen acupoints are selected to tonify kidney essence, regulate the Governor, Conception, and Thoroughfare vessels, and calm the mind, following these therapeutic principles (26). To tonify the kidney, the primary acupoints are Shenshu (BL23) and Taixi (KI3). To regulate the function of the Conception and Thoroughfare vessels, the selected acupoints include Dahe (KI12), Guanyuan (CV4), Zhongwan (CV12), Tianshu (ST25), Zusanli (ST36), and Sanyinjiao (SP6). To calm the mind and regulate emotions, the acupoints Baihui (GV20), Shenting (GV24), Benshen (GB13), and Taichong (LR3) are chosen. Additionally, Ciliao (BL33) and Zigong (EX-CA1) are utilized for their unique therapeutic effects on pelvic diseases and ovarian dysfunction. The treatment focuses on restoring the imbalance of the “emotional (brain) – kidney – Tian Gui – Conception and Thoroughfare vessels – uterus” reproductive axis, with the primary therapeutic principles being “regulating the Conception and Thoroughfare vessels, calming the mind, and tonifying the liver and kidney.” This combination of acupoints addresses both the physiological and emotional aspects of gynecological conditions, aiming to restore balance, improve ovarian function, and regulate emotional well-being (40).

The decreased ovarian function negatively affects women’s mental health, increasing the risk of anxiety and depression (6). Previous studies have revealed that acupuncture has tremendous potential for improving ovarian function, adjusting hormonal levels, elevating mood, and improving quality of life (41). Acupuncture points stimulate the central nervous system, triggering biochemical changes that regulate brain-body functions and promote both physical and emotional well-being (42). The concept of “brain-X axis” has become a prominent research focus in recent years, exploring the complex interactions between the brain and various bodily systems, including the brain-gut axis, brain-bone axis and brain-endocrine axis etc (43, 44). Depression and excessive stress can disrupt the hypothalamic-pituitary-gonadal (HPG) axis, interfering with hormone secretion and leading to issues such as irregular menstruation and reduced libido (44). Deng’s study found that acupuncture at GV20 has been shown to (i) regulate the default mode network and (ii) enhance functional connectivity between the posterior central gyrus, prefrontal cortex, and bilateral anterior cingulate gyrus in patients with depression (45). Pei’s study on POI found that pre-treatment POI patients had significantly higher SAS and Kupperman Index scores than HCs, with improvements in FSH, LH, SAS/Kl scores, and AFC after treatment. This study will conduct a relatively comprehensive observation of various clinical symptoms. The SAS, SDS and SRSS will be used for patient self-assessment of anxiety, depression, and sleep problems, respectively. The combined application of these three scales can provide a more holistic evaluation of mental health status. FC analysis showed increased connectivity between the hypothalamus and various brain regions after acupuncture, while connectivity with the left gyrus rectus decreased (46). Here in this study, we design to use fMRI to estimate brain function and the relationship between brain-emotional and ovarian function related hormones.

The hypothalamus functions as the central command center of the neuroendocrine system, with the HPO axis serving as the dominant regulatory pathway for female reproductive function. This axis precisely controls follicular development and steroid hormone production through gonadotropin-releasing hormone (GnRH)-mediated pulsatile secretion of follicle-stimulating hormone (FSH) and LH (47). Emerging evidence indicates that HPO axis dysregulation, a characteristic feature of diminished ovarian reserve (DOR), manifests not only as reproductive dysfunction but also involves extensive neural network alterations encompassing cortical and limbic systems, reflecting its unique neuro-reproductive integration. At the molecular level, the kisspeptin/GPR54 signaling system acts as the key upstream regulator of GnRH neurons, with anatomically and functionally distinct populations in the anteroventral periventricular nucleus (AVPV) and arcuate nucleus (ARC) mediating estrogen’s positive and negative feedback effects, respectively (48). Importantly, chronic stress-induced activation of the hypothalamic-pituitary-adrenal (HPA) axis leads to sustained glucocorticoid elevation, which directly suppresses GnRH pulsatility and consequently disrupts HPO axis function, establishing a critical pathway through which psychological stress impacts reproductive health (49). In this study, we will use fMRI to analyze hypothalamus and further more in periventricular nucleus such as AVPV and ARC.

This study adopts an interdisciplinary approach (integrating acupuncture, neuroimaging, and gynecology) to elucidate the central neural mechanisms of acupuncture in improving DOR, establishing a paradigm for modernizing research on traditional therapies. Using fMRI, we systematically investigate how acupuncture modulates functional connectivity in the hypothalamic network of DOR patients, innovatively revealing potential neural targets underlying acupuncture’s therapeutic effects. Through multidimensional assessments—including ovarian reserve markers (AMH/AFC/FSH), psychological scales (SAS/SDS/SRSS), and neuroimaging data—we demonstrate the synergistic improvement of acupuncture on reproductive, psychological, and neurological functions. Technically, the study employs the Park Sham Device to standardize sham acupuncture procedures, ensuring rigorous experimental design.

There are two limitations to consider. (a) Due to limited funding, it’s a single-center study, we are unable to conduct multi-center, large-scale trials, which may reduce the generalizability and strength of the evidence. However, we will focus on enhancing patient compliance to optimize the quality of the research. (b) The purpose of this study is to investigate the underlying mechanism of this acupuncture point scheme, and therefore, SA was selected to minimize the placebo effect and enhance the scientific rigor of the experimental design to use as a control group. Given the urgent needs of DOR patients, SA may not produce significant clinical effects, as a result, there may be a higher dropout/loss-to-follow-up rate. In future studies, conventional treatments such as HRT or hormone-modulating therapy (HMT) could be incorporated to fulfill ethical considerations and provide additional comparative insights.

## Conclusion

The principles of acupuncture require further exploration through more comprehensive and multidimensional modern research methodologies in order to provide stronger theoretical support for clinical treatments. Despite acupuncture’s historical and clinical use in treating various conditions, including ovarian reserve dysfunction, its mechanisms of action remain not fully understood, particularly in the context of DOR. A deeper investigation into the neurobiological, hormonal, and psychological effects of acupuncture, utilizing advanced techniques such as functional neuroimaging, molecular biology, and clinical trials, is crucial. By enhancing our understanding of how acupuncture influences the central nervous system, hormonal regulation, and ovarian function, this research could lead to more effective and evidence-based acupuncture protocols for DOR. Ultimately, this approach aims to offer better therapeutic solutions for women with DOR, improving both clinical outcomes and quality of life.

## References

[1] Farquhar CM Bhattacharya S Repping S Mastenbroek S Kamath MS Marjoribanks J . Female subfertility. Nat Rev Dis Primers.. (2019) 5:7. doi: 10.1038/s41572-018-0058-8 30679436

[2] Cohen J Chabbert-Buffet N Darai E . Diminished ovarian reserve, premature ovarian failure, poor ovarian responder—a plea for universal definitions. J Assisted Reprod Genet. (2015) 32:1709–12. doi: 10.1007/s10815-015-0595-y, PMID: 26463876 PMC4681731

[3] Greene AD Patounakis G Segars JH . Genetic associations with diminished ovarian reserve: a systematic review of the literature. J Assist Reprod Genet. (2014) 31:935–46. doi: 10.1007/s10815-014-0257-5, PMID: 24840722 PMC4130940

[4] Bunnewell SJ Honess ER Karia AM Keay SD Al Wattar BH Quenby S . Diminished ovarian reserve in recurrent pregnancy loss: a systematic review and meta-analysis. Fertil Steril.. (2020) 113:818–27.e3. doi: 10.1016/j.fertnstert.2019.11.014, PMID: 32145928

[5] Pastore LM Christianson MS Stelling J Kearns WG Segars JH . Reproductive ovarian testing and the alphabet soup of diagnoses: DOR, POR, and FOR. J Assist Reprod Genet. (2018) 35:17–23. doi: 10.1007/s10815-017-1058-4, PMID: 28971280 PMC5758472

[6] Huang S Zhang D Shi X Zhang Y Wang X She Y . Acupuncture and related therapies for anxiety and depression in patients with premature ovarian insufficiency and diminished ovarian reserve: a systematic review and meta-analysis. Front Psychiatry. (2024) 15:1495418. doi: 10.3389/fpsyt.2024.1495418, PMID: 39687777 PMC11647530

[7] Khalesi ZB Kenarsari FJ . Anxiety, depression, and stress: a comparative study between couples with male and female infertility. BMC Womens Health. (2024) 24:228. doi: 10.1186/s12905-024-03072-5, PMID: 38589804 PMC11003146

[8] Huang Y Cheng Y Zhang M Xia Y Chen X Xian Y . Oxidative stress and inflammatory markers in ovarian follicular fluid of women with diminished ovarian reserve during *in vitro* fertilization. J Ovarian Res. (2023) 16:206. doi: 10.1186/s13048-023-01293-0, PMID: 37872635 PMC10591385

[9] Cai XF Wang BY Zhao JM Nian MX Lin QC Huang JF . Association of sleep disturbances with diminished ovarian reserve in women undergoing infertility treatment. Sci Rep. (2024) 14:26279. doi: 10.1038/s41598-024-78123-w, PMID: 39487261 PMC11530423

[10] Benetti-Pinto CL de Almeida DM Makuch MY . Quality of life in women with premature ovarian failure. Gynecol Endocrinol. (2011) 27:645–9. doi: 10.3109/09513590.2010.520374, PMID: 21214499

[11] Geng X He Z Bao Z Di W Gu Z . Aberrant HPO Axis Alterations and Autoimmune Abnormalities in PCOS Patients with DOR: A Retrospective Analysis. J Clin Med. (2023) 12. doi: 10.3390/jcm12165212, PMID: 37629254 PMC10455465

[12] Mohammed S Sundaram V Adidam Venkata CR Zyuzikov N . Polycystic ovary rat model exposure to 150 kHz intermediate frequency: hypothalamic-pituitary-ovarian axis at the receptor, cellular, tissue, and hormone levels. J Ovarian Res. (2021) 14:173. doi: 10.1186/s13048-021-00914-w, PMID: 34895279 PMC8665544

[13] Wang Y Yuan Y Meng D Liu X Gao Y Wang F . Effects of environmental, social and surgical factors on ovarian reserve: Implications for age-relative female fertility. Int J Gynaecol Obstet.. (2021) 154:451–8., PMID: 33569772 10.1002/ijgo.13567 PMC8451883

[14] Leistner C Menke A . Hypothalamic-pituitary-adrenal axis and stress. Handb Clin Neurol. (2020) 175:55–64. doi: 10.1016/B978-0-444-64123-6.00004-7, PMID: 33008543

[15] Zhang Y Li KS Liu HW Fu CH Chen S Tan ZJ . Acupuncture treatment modulates the resting-state functional connectivity of brain regions in migraine patients without aura. Chin J Integr Med. (2016) 22:293–301. doi: 10.1007/s11655-015-2042-4, PMID: 25847772

[16] Peng W Xu H Zhang C Hu Y Yu S . The altered hypothalamic network functional connectivity in chronic insomnia disorder and regulation effect of acupuncture: a randomized controlled neuroimaging study. BMC Complement Med Ther. (2024) 24:396. doi: 10.1186/s12906-024-04703-y, PMID: 39543627 PMC11566913

[17] Wong KKL Xu J Chen C Ghista D Zhao H . Functional magnetic resonance imaging providing the brain effect mechanism of acupuncture and moxibustion treatment for depression. Front Neurol. (2023) 14:1151421. doi: 10.3389/fneur.2023.1151421, PMID: 37025199 PMC10070747

[18] Yin J Chang H-M Li R Leung PCK . Recent progress in the treatment of women with diminished ovarian reserve. Gynecology Obstetrics Clin Med. (2021) 1:186–9. doi: 10.1016/j.gocm.2021.10.004

[19] Liu Y Ma L Yang X Bie J Li D Sun C . Menopausal Hormone Replacement Therapy and the Risk of Ovarian Cancer: A Meta-Analysis. Front Endocrinol (Lausanne).. (2019) 10:801., PMID: 31849838 10.3389/fendo.2019.00801 PMC6902084

[20] Herlihy NS Cakiroglu Y Whitehead C Reig A Tiras B Scott RT Jr. . Effect of intraovarian platelet-rich plasma injection on IVF outcomes in women with poor ovarian response: the PROVA randomized controlled trial. Hum Reprod. (2024) 39:1495–503. doi: 10.1093/humrep/deae093, PMID: 38725194

[21] Vahabi Dastjerdi M Sheibani S Taheri M Hezarcheshmeh FK Jahangirian J Jazayeri M . Efficacy of intra-ovarian injection of autologous platelet-rich plasma in women with poor responders: a systematic review and meta-analysis. Arch Gynecol Obstet.. (2024) 309:2323–38. doi: 10.1007/s00404-024-07442-0, PMID: 38589612

[22] Wu Y Xiao Q Wang S Xu H Fang Y . Effectiveness of acupuncture for infertility in patients with polycystic ovary syndrome: a systematic review and network meta-analysis. Endocr Metab Immune Disord Drug Targets. (2024). doi: 10.2174/0118715303297819240826065755, PMID: 39313899 PMC13523171

[23] Guven PG Cayir Y Borekci B . Effectiveness of acupuncture on pregnancy success rates for women undergoing *in vitro* fertilization: A randomized controlled trial. Taiwan J Obstet Gynecol.. (2020) 59:282–6., PMID: 32127151 10.1016/j.tjog.2020.01.018

[24] Zhang J Wu X Nie D Zhuo Y Li J Hu Q . Magnetic Resonance Imaging Studies on Acupuncture Therapy in Depression: A Systematic Review. Front Psychiatry. (2021) 12:670739. doi: 10.3389/fpsyt.2021.670739, PMID: 34489749 PMC8417590

[25] Lu G Y-y Z Li H-x Yin Y-l Shen J Shen M-h . Effects of acupuncture treatment on microRNAs expression in ovarian tissues from Tripterygium glycoside-induced diminished ovarian reserve rats. Front Genet. (2022) 13. doi: 10.3389/fgene.2022.968711, PMID: 36212128 PMC9532950

[26] Xiao Q Wu Y Su C Yang J Wang J Pei L . Exploring the efficacy and safety of acupuncture versus sham acupuncture for diminished ovarian reserve: study protocol for a multicentre randomised controlled trial. BMJ Open. (2024) 14. doi: 10.1136/bmjopen-2023-081098, PMID: 39160098 PMC11337660

[27] Zhu H Nan S Suo C Zhang Q Hu M Chen R . Electro-Acupuncture Affects the Activity of the Hypothalamic-Pituitary-Ovary Axis in Female Rats. Front Physiol. (2019) 10:466. doi: 10.3389/fphys.2019.00466, PMID: 31068836 PMC6491808

[28] Bai T Zhou E Wang K Li W Bi J Ju J . Study protocol for a randomized controlled trial: evaluating the impact of acupuncture on menstrual regulation and pregnancy enhancement in patients with DOR using rs-fmri to assess brain functional networks. J Multidiscip Healthcare. (2024) 17:5425–34. doi: 10.2147/JMDH.S490162, PMID: 39582877 PMC11586002

[29] Kim J Kim S-R Lee H Nam D-H . Comparing verum and sham acupuncture in fibromyalgia syndrome: A systematic review and meta-analysis. Evidence-Based Complementary Altern Med. (2019) 2019:1–13. doi: 10.1155/2019/8757685, PMID: 31534469 PMC6732586

[30] Vickers AJ Linde K . Acupuncture for chronic pain. Jama. (2014) 311:955–6. 10.1001/jama.2013.285478 PMC4036643 24595780

[31] Xu S Yu L Luo X Wang M Chen G Zhang Q . Manual acupuncture versus sham acupuncture and usual care for prophylaxis of episodic migraine without aura: multicentre, randomised clinical trial. Bmj. (2020) 368:m697. doi: 10.1136/bmj.m697, PMID: 32213509 PMC7249245

[32] Usichenko TI Wesolowski T Lotze M . Verum and sham acupuncture exert distinct cerebral activation in pain processing areas: a crossover fMRI investigation in healthy volunteers. Brain Imaging Behav. (2015) 9:236–44. doi: 10.1007/s11682-014-9301-4, PMID: 24728839

[33] To M Alexander C . The effects of Park sham needles: a pilot study. J Integr Med. (2015) 13:20–4. doi: 10.1016/S2095-4964(15)60153-4, PMID: 25609368

[34] Lund I Lundeberg T . Are Minimal, Superficial Or Sham Acupuncture Procedures Acceptable as Inert Placebo Controls? Acupuncture Med. (2006) 24:13–5., PMID: 16618044 10.1136/aim.24.1.13

[35] Zhang L Tang Y Hui R Zheng H Deng Y Shi Y . The effects of active acupuncture and placebo acupuncture on insomnia patients: a randomized controlled trial. Psychology Health Med. (2020) 25:1201–15. doi: 10.1080/13548506.2020.1738015, PMID: 32167794

[36] Desmond JE Glover GH . Estimating sample size in functional MRI (fMRI) neuroimaging studies: Statistical power analyses. J Neurosci Methods. (2002) 118:115–28. doi: 10.1016/S0165-0270(02)00121-8, PMID: 12204303

[37] Skorupskaite K George JT Anderson RA . The kisspeptin-GnRH pathway in human reproductive health and disease. Hum Reprod Update.. (2014) 20:485–500., PMID: 24615662 10.1093/humupd/dmu009 PMC4063702

[38] Ogawa A Osada T Tanaka M Kamagata K Aoki S Konishi S . Connectivity-based localization of human hypothalamic nuclei in functional images of standard voxel size. Neuroimage. (2020) 221:117205., PMID: 32735999 10.1016/j.neuroimage.2020.117205

[39] Yani XU Yutong Z Weile HE Linglin D Ding T Jialing W . Efficiency and safety of acupuncture for women with premature ovarian insufficiency: study protocol for a randomized controlled trial. J Tradit Chin Med. (2023) 43:1268–74., PMID: 37946490 10.19852/j.cnki.jtcm.20230214.002 PMC10623257

[40] Yang L Xu HF Gou MH Yang HS Feng YX Liu SY . Application of Tiaojing Cuyun acupuncture in treatment of diseases with ovarian function decline. Zhongguo Zhen Jiu.. (2022) 42:1200–4., PMID: 37199213 10.13703/j.0255-2930.20220505-k0002

[41] Eisenhardt S Fleckenstein J . Traditional Chinese medicine valuably augments therapeutic options in the treatment of climacteric syndrome. Arch Gynecol Obstet.. (2016) 294:193–200., PMID: 27040419 10.1007/s00404-016-4078-x

[42] Wong KKL Xu J Chen C Ghista D Zhao H . Functional magnetic resonance imaging providing the brain effect mechanism of acupuncture and moxibustion treatment for depression. Front Neurology.. (2023) 14:1151421., PMID: 37025199 10.3389/fneur.2023.1151421 PMC10070747

[43] Li R Miao Z Liu Y Chen X Wang H Su J . The Brain-Gut-Bone Axis in Neurodegenerative Diseases: Insights, Challenges, and Future Prospects. Adv Sci (Weinh).. (2024) 11:e2307971. doi: 10.1002/advs.202307971, PMID: 39120490 PMC11481201

[44] Mbiydzenyuy NE Qulu L-A . Stress, hypothalamic-pituitary-adrenal axis, hypothalamic-pituitary-gonadal axis, and aggression. Metab Brain Disease.. (2024) 39:1613–36., PMID: 39083184 10.1007/s11011-024-01393-w PMC11535056

[45] Deng D Liao H Duan G Liu Y He Q Liu H . Modulation of the Default Mode Network in First-Episode, Drug-Naïve Major Depressive Disorder via Acupuncture at Baihui (GV20) Acupoint. Front Hum Neurosci. (2016) 10:230. doi: 10.3389/fnhum.2016.00230, PMID: 27242492 PMC4869560

[46] Zhao TT Liu YK Zhou JL Ning HX Wu XL Song YF . Effect of acupuncture on hypothalamic functional connectivity in patients with premature ova-rian insufficiency based on resting-state functional magnetic resonance imaging. Zhen Ci Yan Jiu.. (2022) 47:617–24., PMID: 35880279 10.13702/j.1000-0607.20210399

[47] Mikhael S Punjala-Patel A Gavrilova-Jordan L . Hypothalamic-Pituitary-Ovarian Axis Disorders Impacting Female Fertility. Biomedicines. (2019) 7. doi: 10.3390/biomedicines7010005, PMID: 30621143 PMC6466056

[48] McEwen BS Milner TA . Understanding the broad influence of sex hormones and sex differences in the brain. J Neurosci Res. (2017) 95:24–39. doi: 10.1002/jnr.v95.1-2, PMID: 27870427 PMC5120618

[49] Valsamakis G Chrousos G Mastorakos G . Stress, female reproduction and pregnancy. Psychoneuroendocrinology. (2019) 100:48–57. 30291988 10.1016/j.psyneuen.2018.09.031

