# Correction: The altered hypothalamic network functional connectivity in diminished ovarian reserve and regulation effect of acupuncture: a randomized controlled neuroimaging study protocol

**DOI:** 10.3389/fendo.2025.1734258

**Published:** 2025-11-14

**Authors:** Feng Gao, Yang Yu, Fuwei Wang, Yigong Fang

**Affiliations:** 1 Institute of Acupuncture and Moxibustion, China Academy of Chinese Medical Sciences (CACMS), Beijing, China; 2 Department of Acupuncture and Tuina, School of Medicine of the Affiliated Sir Run Run Shaw Hospital, Zhejiang University, Hangzhou, China

**Keywords:** diminished ovarian reserve, rs- fMRI, hypothalamic-pituitary-ovarian axis, functional connectivity, acupuncture

Author “Yigong Fang” was erroneously assigned to affiliation 2. “Department of Acupuncture and Tuina, School of Medicine of the Affiliated Sir Run Run Shaw Hospital, Zhejiang University, Hangzhou, China”. The author was only affiliated to Affiliation: “Institute of Acupuncture and Moxibustion, China Academy of Chinese Medical Sciences (CACMS), Beijing, China”.

The authors names were erroneously swapped around. Instead of “Gao Feng, Yu Yang, Wang Fuwei, and Fang Yigong”, it should be “Feng Gao, Yang Yu, Fuwei Wang, and Yigong Fang".

And lastly, the funder “Supported by: Scientific and Technological Innovation Project of China Academy of Chinese Medical Sciences (Grant Number: CI2021B012)” to Yigong Fang was erroneously omitted.

The original version of this article has been updated.

